# Pronounced Effects of Acute Endurance Exercise on Gene Expression in Resting and Exercising Human Skeletal Muscle

**DOI:** 10.1371/journal.pone.0051066

**Published:** 2012-11-30

**Authors:** Milène Catoire, Marco Mensink, Mark V. Boekschoten, Roland Hangelbroek, Michael Müller, Patrick Schrauwen, Sander Kersten

**Affiliations:** 1 Division of Human Nutrition, Wageningen University, Wageningen, The Netherlands; 2 Netherlands Nutrigenomics Centre, Top Institute Food and Nutrition, Wageningen, The Netherlands; 3 Department of Human Biology, Maastricht University Medical Centre, Maastricht, The Netherlands; University of Sydney, Australia

## Abstract

Regular physical activity positively influences whole body energy metabolism and substrate handling in exercising muscle. While it is recognized that the effects of exercise extend beyond exercising muscle, it is unclear to what extent exercise impacts non-exercising muscles. Here we investigated the effects of an acute endurance exercise bouts on gene expression in exercising and non-exercising human muscle. To that end, 12 male subjects aged 44–56 performed one hour of one-legged cycling at 50% W_max_. Muscle biopsies were taken from the exercising and non-exercising leg before and immediately after exercise and analyzed by microarray. One-legged cycling raised plasma lactate, free fatty acids, cortisol, noradrenalin, and adrenalin levels. Surprisingly, acute endurance exercise not only caused pronounced gene expression changes in exercising muscle but also in non-exercising muscle. In the exercising leg the three most highly induced genes were all part of the NR4A family. Remarkably, many genes induced in non-exercising muscle were PPAR targets or related to PPAR signalling, including PDK4, ANGPTL4 and SLC22A5. Pathway analysis confirmed this finding. In conclusion, our data indicate that acute endurance exercise elicits pronounced changes in gene expression in non-exercising muscle, which are likely mediated by changes in circulating factors such as free fatty acids. The study points to a major influence of exercise beyond the contracting muscle.

## Introduction

Regular exercise training is generally recognized as a powerful preventive and therapeutic strategy for diseases such as type 2 diabetes, obesity and cardiovascular disease. At a systemic level, regular exercise training improves cardiac and lung function [1], [2], [3], reduces the amount of adipose tissue [4], increases muscle mass [5], [6], [7], and decreases liver fat [8], [9], representing chronic adaptations to repeated exercise bouts. Interestingly, the observation that unilateral training also improves strength in the immobilized or untrained limb indicates that the beneficial effects of exercise are not limited to the tissues directly engaged in exercise [10], [11], [12].

Immediately upon initiation of exercise, local demand for ATP, oxygen, glucose and fatty acids increases dramatically. These demands are accommodated by rapid changes in skeletal muscle activity of key enzymes and transporters involved in glucose and fatty acid oxidation via allosteric regulation and phosphorylation of rate-limiting enzymes. In addition, regulation at the mRNA level importantly contributes to the acute response and chronic adaptations to exercise. A large number of studies have shown that acute exercise induces genes involved in a variety of processes, including energy metabolism, hypertrophy and signalling [13], [14], [15], [16], [17], [18], [19], [20], [21]. Whole genome mRNA profiling has confirmed these findings, revealing major changes in skeletal muscle gene expression from 1 hour to even 48 hours after cessation of exercise [22], [23], [24], [25]. All efforts to characterize exercise-induced changes in mRNA have so far focused on the exercising muscle. To what extent exercise influences gene expression in non-exercising muscles remains completely unclear. Conceivably, exercise may elicit changes in gene expression in non-exercising muscle via circulating mediators and metabolites. Such a mechanism may provide a conceptual framework for the impact of exercise on non-contractile tissues such as liver. In the present study, we have employed the one-legged exercise model and pre- and post-exercise muscle biopsies to study the acute effects of exercise on whole genome gene expression in exercising and resting human skeletal muscle. The results reveal that acute endurance exercise elicits pronounced changes in gene expression in non-exercising muscle, which are likely mediated by changes in circulating factors such as free fatty acids (FFA).

## Methods

### Subjects

Twelve healthy middle-aged men (age 51.5±5.1 years, body weight 88±17 kg, body mass index 26±4) participated in the study. All subjects exercised less than 4 hours per week. Anthropometric parameters, VO_2max_ and W_max_ (1 and 2 legged) values can be found in table 1. The study was approved by the medical ethical committee of Wageningen University and all subjects received oral and written information about the experimental procedures and provided written informed consent.

**Table 1:** Subject characteristics (N = 12).

Subject characteristics (N = 12)	
Age (years)	52±5
Length (cm)	184±5
Weight (kg)	88±17
BMI (kg/m2)	26±4
Rest HR (bpm)	60±11
Maximum HR (bpm)	170±17
VO2MAX (ml/min/kg)	35±10
VO2Peak 1 leg (ml/min/kg)	28±10
Wmax 1 leg (watts)	164±37
Wmax 2 legs (watts)	260±64

Values are mean ± standard deviation. HR  =  heart rate, BMI  =  body mass index, VO2MAX  =  maximum oxygen uptake, VO2Peak  =  peak oxygen uptake, Wmax  =  maximum work load.

### Experimental design

**Figure 1:**
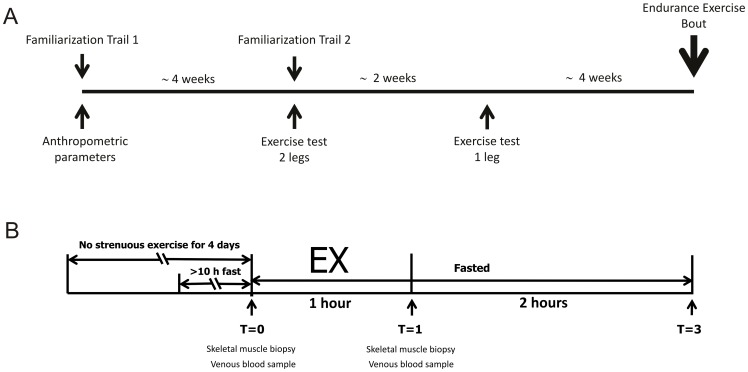
Experimental design. The timeline of the study (A) and set-up of the endurance exercise bout (B). After 2 familiarization trails and 2 exercise tests, subjects performed 1 hour of submaximal one-legged endurance exercise. Before and after the exercise bout muscle biopsies and venous blood samples were taken, and another blood sample was taken 2 hours after the end of exercise.

All subjects performed a single 60 minutes experimental endurance exercise bout, which was preceded by two preliminary exercise tests and two familiarization trials (figure 1A). During the endurance exercise bout subjects had to perform one-legged cycling on a cycle ergometer (Excalibur Sport, Lode, Groningen NL) adapted with a custom-made leg support. Skeletal muscle biopsies were taken from both legs immediately before and shortly after exercise.

### Preliminary exercise tests

Two graded cycling exercise tests to exhaustion were performed, the first with both legs, the second with one leg (Excalibur Sport, Lode, Groningen NL). The first two-legged exercise test was used to determine the subjects' maximum aerobic capacity (VO_2_max; table 1), while the second test was used to determine maximum workload of the dominant experimental leg (Wmax-1-leg; table 1). Both tests started at a set workload (100 watt 2 legs, 20 watt 1 leg), which increased gradually until exhaustion (15 watts per minute 2 legs, 10 watts per minute 1 leg), determined as the participant not being able to continue cycling at 60 rounds per minute for longer than 15 seconds. Respiratory quotient (RQ) was above 1 at the end of the test in all subjects. During the tests oxygen uptake (VO_2_) and heart rate (HR) were measured (Oxycon Pro, Jaeger, Hoechberg, Germany). The tests were performed at the same ergometer as the endurance exercise bout and familiarization trails and were completed at least 14 days before the endurance exercise bout and 3 days apart to avoid a training effect. The day prior to both tests subjects were asked to refrain from alcohol and heavy exercise.

### Familiarization trials

All participants were unfamiliar with one-legged exercise. Therefore the two familiarization trials were performed before the endurance exercise bout to make sure that subjects became familiar with one-legged cycling (figure 1A). The familiarization trials consisted of 20 minutes of one-legged cycling at a self-chosen workload. All subjects performed the one-legged exercise with the dominant leg, which was determined via a number of daily life related questions.

### Experimental endurance exercise bout

Subject refrained from heavy exercise the last 4 days prior to the experimental day. The last day before the experimental exercise bout subjects received a standardized evening meal and refrained from alcohol. On the morning (8.15 h) of the experimental test subjects reported to the research facility, after an overnight fast (>10 hours). The experimental exercise bout consisted of 60 min one-legged cycling at 50% of the one-legged Wmax. Before (T0) and shortly after (T1) the exercise a venous blood sample was drawn and muscle biopsies were taken from both legs (see figure 1B). Two hours after cessation of the exercise a third blood sample was taken (T3). During the experimental exercise HR was recorded continuously. Subjects remained fasted until after the last blood sampling, but were allowed to drink water ad libitum.

### Blood samples

Blood was collected in EDTA containing tubes. The samples were immediately centrifuged at 1000 g at 4°C for 10 minutes, after which plasma was stored in −80°C until further analysis. Blood samples were analysed for free fatty acids (Centre for Medical Diagnostics (SHO), Velp, NL), glucose, triglycerides, cortisol, lactate (Gelderse Vallei hospital, Ede, NL), catecholamines (laboratory of clinical chemistry, Radboud Medical Center, Nijmegen, NL), and insulin (enzyme-linked immunosorbent assay, Mercodia, Uppsala, Sweden).

### Muscle biopsies

Percutaneous needle biopsies were taken before (T0) and shortly after (T1) exercise from the vastus lateralis muscle from both legs (4 biopsies in total), using the Bergström technique with suction [26]. Skin was anesthetized with Xylocaine 2% with Adrenaline. All biopsies were taken from a separate incisions. There was at least a 2 cm gap between the biopsies of T0 and T1 to prevent influence of the earlier biopsy. The second biopsy from the same leg was taken from a more proximal position. Pre-exercise biopsies were taken just before the exercise; post-exercise biopsies within 30 minutes after termination of the exercise bout, on average it took ∼15 minutes before the first post-exercise biopsy was taken. Biopsies of the exercising leg were taken first, followed shortly afterwards by the biopsy of the non-exercising leg. After each biopsy, the collected tissue sample was carefully cleared from visible adipose tissue and blood and divided into four pieces. Three pieces were directly frozen into liquid nitrogen and one piece was embedded into Tissue-Tek O.C.T. compound (Sakura Tissue Tek, Alphen a/d Rijn, NL) and frozen in liquid-nitrogen cooled isopentane, and stored at −80°C for further analysis. We were not able to collect muscle biopsies of one of the participants due to hypersensitivity of the participant to the biopsy procedure.

### RNA extraction

Total RNA was isolated from the skeletal muscle tissue by using Trizol reagent (Invitrogen, Breda, NL). Thereafter RNA was purified using the Qiagen RNeasy Micro kit (Qiagen, Venlo, NL) and RNA quality was checked using an Agilent 2100 bioanalyzer (Agilent Technologies, Amsterdam, NL).

### Microarray processing

Total RNA (100 ng) was labelled using an Ambion WT expression kit (Life Technologies, Bleiswijk, The Netherlands) and hybridized to human whole genome Genechip Human Gene 1.1 ST arrays coding 19.732 genes, (Affymetrix, Santa Clara, CA). Sample labelling, hybridization to chips and image scanning was performed according manufacturer's instructions.

### Microarray data analysis

Microarray analysis was performed using MADMAX pipeline for statistical analysis of microarray data [27]. Quality control was performed and all arrays met our criteria, except arrays from 2 participants that showed a clearly distinct clustering and pattern after normalization. Those microarrays were excluded from further analysis. For further analysis a custom annotation was used based on reorganized oligonucleotide probes, which combines all individual probes for a gene [28]. Expression values were calculated using robust multichip average (RMA) method, which includes quantile normalisation [29]. Microarray data were filtered, and probe sets with expression values higher than 20 on more than 5 arrays were considered to be expressed and selected for further statistical analysis. In addition, an Inter Quartile Range (IQR) cut-off of 0.2 was used to filter out genes that showed no variation between the conditions. Significant differences in expression were assessed using Intensity-Based Moderated T-statistic (IBMT [30]). Genes were defined as significantly changed when the P value was <0.01. Differences in gene expression between the legs were determined using a paired IBMT test on the difference between T0 and T1 for both legs (p<0.05).

Two subjects (4 and 8) were classified as outliers based on their aberrant response to exercise in the non-exercising leg. In subject 4 and 8, expression of 6 out of the 10 most highly upregulated genes was higher than the average plus two times the standard deviation. Accordingly, the analysis was repeated without these two subjects.

All microarray data are MIAME compliant and have been submitted to the Gene Expression Omnibus (accession number GSE41769).

### Pathway analysis

Geneset enrichment analysis (GSEA; http://www.broad.mit.edu/gsea/) was performed for both legs using MADMAX and genesets with a false discovery rate (FDR)<0.2 were considered significantly enriched. Possible transcription factors playing a role in the activation and inhibition of genes were identified using Ingenuity Pathway Analysis (IPA; Ingenuity Systems, Redwood City, CA). The ClueGO plugin in Cytoscape was used for gaining insight into processes activated during exercise in both legs [31], [32].

### cDNA synthesis and quantitative real time PCR

Total RNA was reverse transcribed with a cDNA synthesis kit (Promega, Leiden, NL). Standard qPCR was performed using SensiMix real time PCR reagents (Bioline, London, UK) and a Bio-Rad CFX384 machine (Bio-Rad laboratories, Veenendaal, NL). Primer sequences were based on availability in the PRIMERBANK (http://pga.mgh.harvard.edu/primerbank/index.html) and can be found in table 2. qPCR data were normalized using GAPDH as housekeeping gene for the human samples, since it was shown to be stable within skeletal muscle during exercise [33], and was stable between the time points according to our microarray analysis.

**Table 2:** Primer sequences used for qPCR.

Gene Name	Primer Sequence
NR4A1-F	ATGCCCTGTATCCAAGCCC
NR4A1-R	GTGTAGCCGTCCATGAAGGT
NR4A2-F	GTTCAGGCGCAGTATGGGTC
NR4A2-R	AGAGTGGTAACTGTAGCTCTGAG
NR4A3-F	CAGCACTGAGATCACGGCTAC
NR4A3-R	CCCTCCACGAAGGTACTGATG
FOS-F	CACTCCAAGCGGAGACAGAC
FOS-R	AGGTCATCAGGGATCTTGCAG
JUNB-F	CCTACCGGAGTCTCAAAGCG
JUNB-R	CGAGCCCTGACCAGAAAAGTA
GAPDH-F	GAAGGTGAAGGTCGGAGTC
GAPDH-R	GAAGATGGTGATGGGATTTC

### Statistical analysis

Statistical analysis for the plasma parameters and qPCR results were performed using SPSS (version 18, SPSS, Chicago, IL). Differences between the different time points for the plasma parameters (TG, glucose, FFA, lactate, insulin, cortisol, adrenaline and noradrenaline) were determined using a repeated measure one-way ANOVA. Differences between T0 and T1 in qPCR were evaluated using a paired t-test, differences between the legs in qPCR were evaluated using a paired t-test for the changes between T0 and T1 in each leg. Data are mean ± SD and p<0.05 was considered statistically significant.

## Results

### Systemic effects of one-legged exercise

**Figure 2:**
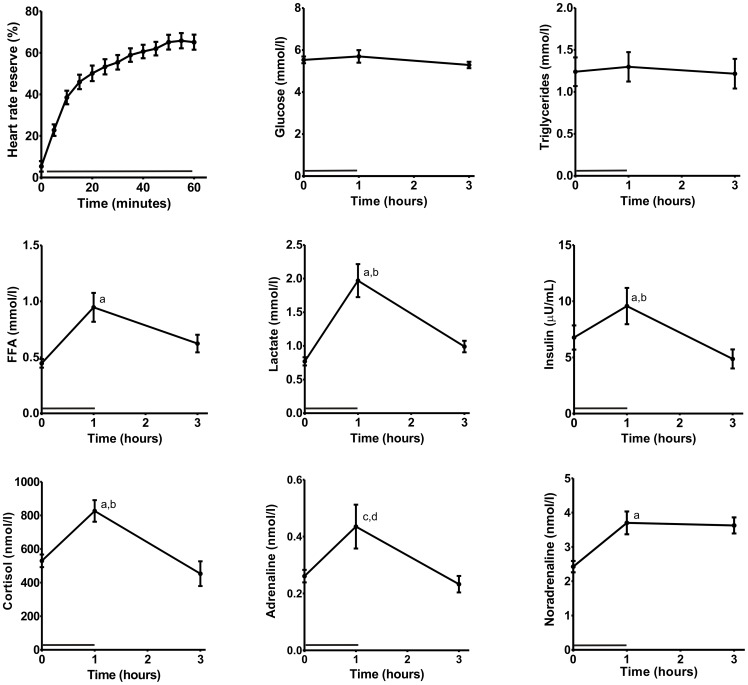
Exercise increases heart rate and plasma levels of FFA, insulin, cortisol and noradrenaline. Heart rate reserve (%) was calculated based on the heart rate measured during the exercise (N = 12). Plasma glucose, triglyceride, free fatty acids, lactate, insulin, cortisol, adrenaline and noradrenaline were measured before and after exercise (T0 and T1; N = 12) and after 2 hours of recovery (T3; N = 12). a = p<0.05 compared to T0, b = p<0.5 compared to T3, c = p<0.1 compared to T0, p<0.1 compared to T3, repeated measures ANOVA. Depicted is mean ± SEM.

Subjects performed one-legged exercise for one hour at 50% of their maximal workload with an average heart rate during the last five minutes of 132±18 beats per minute. The percentage heart rate reserve (%HRR) during the last 5 minutes was 65±12.5% (figure 2). Plasma glucose and triglyceride (TG) levels were not altered by one-legged cycling, while plasma FFA and lactate increased at T3, but mostly returned to baseline at T3 (figure 2). Insulin, cortisol and noradrenalin increased during exercise, with insulin and cortisol returning to baseline at T3 (figure 2). Adrenalin tended to increase during exercise, although the increase was not statistically significant (p = 0.08; figure 2).

### One-legged cycling exercise altered gene expression in skeletal muscle of the exercising and non-exercising leg

We first ruled out that there was a significant difference in baseline expression between the two legs. Only eight out of 19,732 genes were found to be significantly different between the two legs at baseline (p<0.01; table S1).

**Figure 3:**
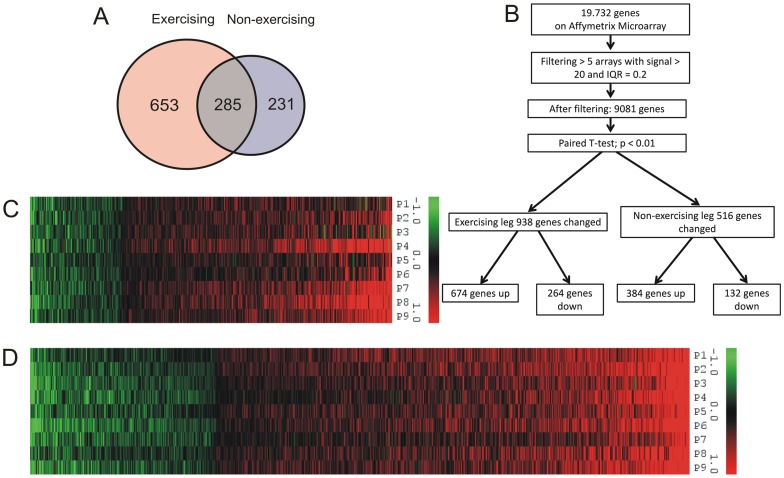
Exercise mainly causes upregulation of gene expression in both the exercising and non-exercising leg. (A) Venn diagram of significantly regulated genes and their overlap. (B) Flowchart of microarray analysis. Heatmaps of all significant genes in the non-exercising (C) and exercising leg (D) N = 9, IQR  =  interquartile range.

Statistical comparison of the baseline and post-exercise samples revealed that in the exercising leg (E) one-legged exercise significantly changed expression of 938 genes (p<0.01), with the majority of genes being upregulated (figure 3A and D). The number of genes significantly changed by exercise in the non-exercising leg (NE) was lower but still remarkably high (p<0.01; 516 genes), and also here the majority of genes was upregulated (figure 3A and C). Intriguingly, the majority of genes altered in the non-exercising leg were also altered in the exercising leg (figure 3A). Overall, the data indicate that a single exercise bout of exercise caused marked changes in gene expression not only in exercising muscle, but also in non-exercising muscle and that exercise mainly promotes upregulation of gene expression.

**Figure 4:**
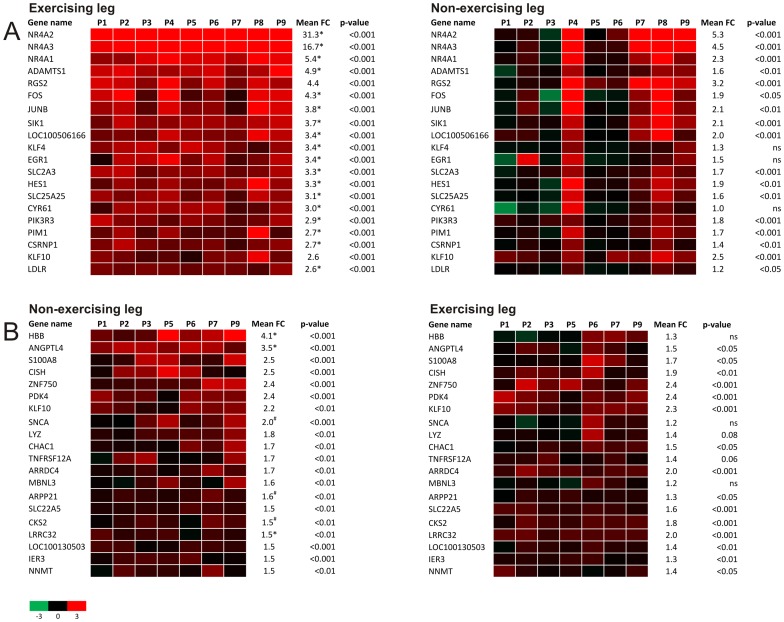
Top 20 of most highly induced genes in exercising and non-exercising leg. A) Left panel shows the top 20 of upregulated genes in the exercising leg (N = 9), right panel the corresponding genes in the non-exercising leg. B) Left panel shows the top 20 of upregulated genes in the non-exercising leg (N = 7), right panel the corresponding genes in the exercising leg. Green is a signal log ratio of −3, red a signal log ratio of 3. Values are displayed per subject to visualize inter-individual differences. FC = fold change, *  = p<0.05, ^#^  = p<0.1 between exercising and non-exercising leg.

To gain more insight into the changes induced by exercise, genes were subsequently ranked according to mean fold-change in expression in the exercising leg and the changes in expression compared between the individual subjects (figure 4A, left panel). Expression changes of the same set of genes in the non-exercising leg are presented in parallel (figure 4A, right panel). Remarkably, the most highly induced genes in the exercising leg are the three members of the nuclear receptor subfamily NR4A. Of the 20 most highly induced genes in the exercising leg, 17 were also significantly upregulated in the non-exercising leg although to a lesser extent. Figure 4A clearly illustrates the marked inter-individual variation in response to exercise in the non-exercising leg. qPCR of selected genes largely confirmed the results of the microarray (figure S1).

To examine the impact of exercise on gene expression in parts of the body not directly influenced by exercise, we next focused our attention on genes upregulated in the non-exercising leg. Similarly to the exercising leg, genes were ranked according to mean fold-change in the non-exercising leg (figure S2, left panel) and changes in the exercising leg are shown in parallel (figure S2, right panel). Many of the most highly upregulated genes in the non-exercising leg were also highly induced in the exercising leg (figure S2). The two most highly upregulated genes were again NR4A2 (FC  = 5.3) and NR4A3 (FC  = 4.5). Importantly, subject 4 and 8 both showed a distinct profile from the other subjects illustrated by marked induction of numerous genes in the non-exercising leg that are not shared with other subjects. Based on the notion that gene expression was clearly distinctive from other subjects and using specific criteria outlined in the methods, we classified them as outliers and repeated statistical analysis without subject 4 and 8. We suspect that these subjects engaged in involuntary (isometric) contractions or other type of stimulation of the non-exercising leg. Removal of both subjects markedly reduced the number of significantly changed genes (209 vs. 516 genes). Furthermore, the overlap in gene regulation between the exercising and non-exercising leg decreased from 285 to 85 genes. After removing subject 4 and 8, many of the most highly upregulated genes in the non-exercising leg were characterized by lower induction in the exercising leg. Other genes showed similar fold-induction in the exercising and non-exercising leg, including ZNF750, PDK4, KLF10, and SLC22A5 (figure 4B), which are established or suspected target genes of PPARs [34].

### Processes during one legged cycling in the exercising leg and non-exercising leg

**Figure 5:**
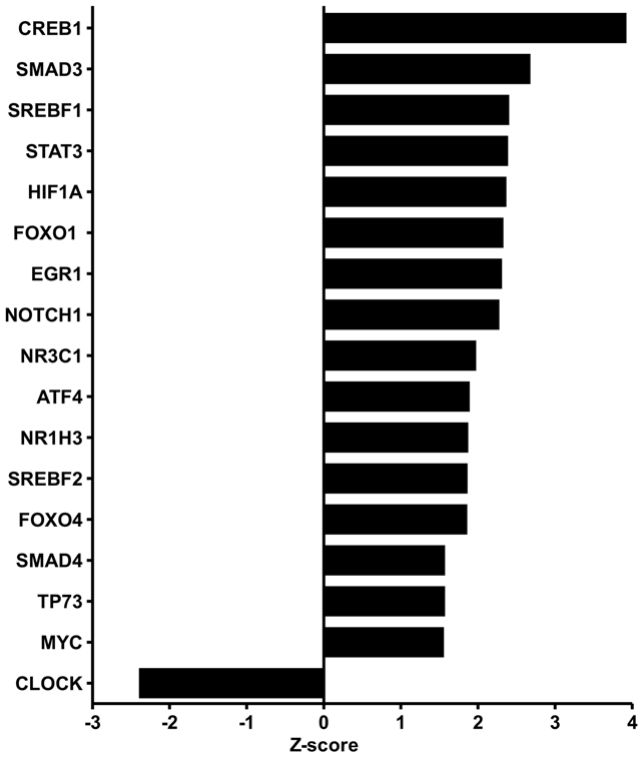
Induction of transcription factor pathways by exercise. Transcription factor pathways related to growth, stress response, cAMP signalling and hypoxia were induced by exercise. Transcription factor pathways were identified for the exercising leg using IPA and are displayed in a bar diagram. Genes induced by exercise for the different transcription factors can be found in table S1. Transcription factors with a z-score above 1.5 (or under −1.5) are considered as biologically relevant.

We suspected that exercise-induced changes in gene expression were mediated by specific transcription factors. To identify these transcription factors and to identify pathways regulated by exercise, we performed Ingenuity pathway analysis (IPA) (figure 5 and table S2). IPA uses information from literature combined with gene expression changes to predict a role of transcription factors in the dataset. The most significant set of target genes in the exercising leg was the set controlled by CREB1. CREB1 and also ATF4 (lower in the list) are both induced by cAMP and mediate cAMP-dependent gene regulation. SREBF1, SREBF2 and NR1H3 (LXR) are all involved in lipid and especially cholesterol homeostasis and have extensive cross-talk. Other sets of target genes shown in figure 5 are involved in growth (sets under control of STAT3, FOXO1, NOTCH1, MYC and NR3C1) and inflammation (sets under control of NR3C1 and STAT3). HIF1A is a major regulator in the adaptive responses to hypoxia.

The only significant set of target genes in the non-exercising leg was the set controlled by PPARα, which is consistent with the marked upregulation of several existing and putative PPARα target genes (table S2). Since PPARα is activated by fatty acids [35], these data point to a role of elevated plasma FFA levels in exercise-induced changes in the non-exercising leg, and to a lesser extent in the exercising leg, as other processes seem to play a more pronounced role there.

**Figure 6:**
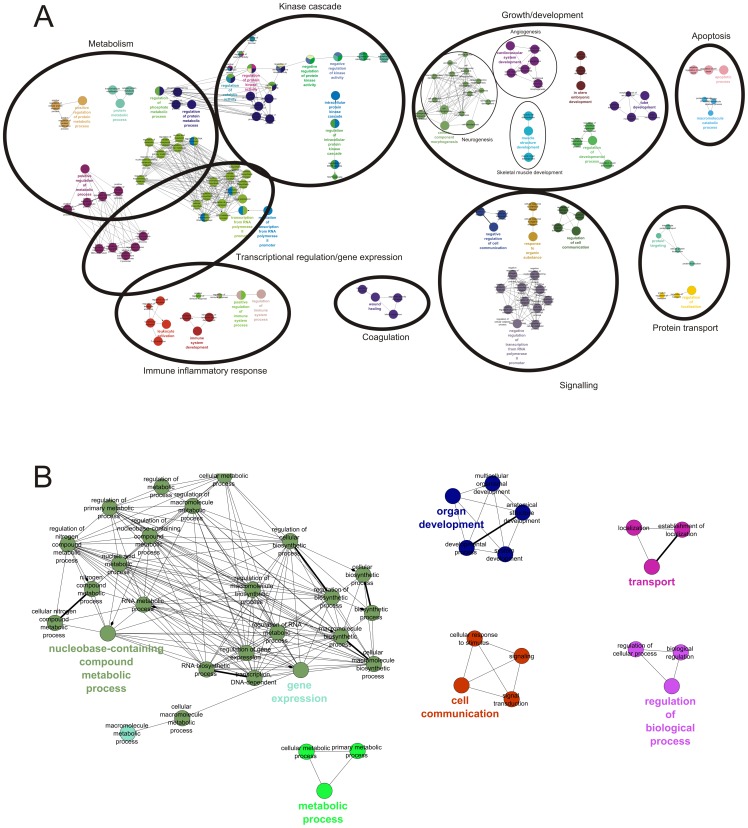
ClueGO network analysis. Analysis shows significant regulation of several GO categories involved in skeletal muscle development, angiogenesis, inflammation and MAPK cascade in the exercising leg (A; N = 9) and basal metabolism and signalling in the non-exercising leg (B; N = 7). The nodes represent significantly changed GO categories. Lines represent the overlap between different categories. All nodes with a large overlap have a similar colour.

To further determine the biological processes activated during exercise, we used ClueGO and GSEA. ClueGO integrates GO categories and creates a functionally organized GO category networks based on the overlap between the different GO categories and the significance [31]. According to ClueGO analysis, a substantial number of processes was induced during exercise in both legs (figure 6). In the exercising leg the important processes were growth and development of skeletal muscle, neurons and vessels, metabolism (mostly basal and protein metabolism) and transcriptional regulation (figure 6A). Also kinase cascade and signalling were induced. GSEA showed an comparable picture, with upregulation of genesets involved in growth (hypertrophy model), MAPK signalling and stress response (AP1 pathway; figure S3). ClueGO revealed that most processes induced by exercise in the non-exercising leg are involved in basal metabolism and signalling/transport (figure 6B). GSEA showed a clear upregulation of PPAR target genes, (cytokine) signalling and growth and stress response (hypertrophy & AP1 pathway; figure S3).

## Discussion

Here we used the one-legged exercise model to study the effect of acute endurance exercise on whole genome muscle gene expression and determine the relative importance of systemic versus local contraction-related stimuli. We found that acute exercise induced immediate and dramatic gene expression changes in the exercising leg, with the most dramatic inductions observed for the NR4A family. Strikingly, acute exercise also caused substantial gene expression changes in non-exercising leg. Overall, our data indicate that the molecular responses to acute exercise are not confined to the exercising muscle but also extend to resting muscle. The notion that exercise alters gene expression in non-exercising muscles is new. Our data provide a conceptual and molecular framework for the observation that immobilized muscle can experience favourable metabolic adaptations in response to repeated contractile activity in non-immobilized muscle [10], [11], [12].

In general, changes in gene expression in non-exercising muscle were less pronounced compared to the exercising muscle, both in magnitude of fold-changes and number of genes changed. A relatively small number of genes, many of which represent known target genes of the PPAR transcription factors including ANGPTL4, KLF10, SLC22A5, ZNF750, PDK4, was induced equally in exercising and non-exercising muscle. PPARs play a key role in regulation of lipid metabolism in a variety of tissue, including skeletal muscle [36]. Ingenuity pathway analysis and GSEA further indicated significant enrichment of PPAR target genes in set of genes upregulated in the non-exercising leg. Induction of PPARα targets is expected to lead to enhanced fatty acid oxidation as an acute but perhaps also adaptive response to endurance exercise. Inasmuch as plasma FFA levels go up during exercise and fatty acids are known ligands of PPARs and ANGPTL4 and PDK4 [37], [38], our data may point to an important role of elevated FFAs as systemic factor driving gene expression changes in exercising and non-exercising muscle during exercise. One gene (ANGPTL4) was more highly induced in the non-exercising leg compared to the exercising leg. Detailed exploration of the regulation and role of ANGPTL4 during exercise will be reserved for a future publication. Apart from FFAs, it could be hypothesized that other factors including other metabolites and circulating hormones may also impact gene expression in non-exercising muscle. NR4A transcription factors are known to be regulated via β-adrenergic signalling [39]. Elevated catecholamines (via sympathetic innervation or via the circulation) may at least partially account for the induction of NR4A genes in the exercising leg, which was supported by Ingenuity Pathway Analysis showing the CREB1 pathway as most significant transcription factor pathway. However, even though circulating levels of catecholamines were increased, expression of NR4A genes was not increased in the non-exercising leg, indicating only a minor role of catecholamines in the non-exercising leg. It can be hypothesized that gene expression changes in the non-exercising leg may also be elicited by myokines secreted from the exercising leg. In a future publication we will address the impact of exercise on secretion of myokines using a so called secretome approach followed by measurement of numerous potentially novel and existing myokines in plasma. However, this type of analysis is beyond the scope of the present manuscript.

We are the first to use microarray to assess the immediate effect of acute endurance exercise on human skeletal muscle gene expression. Prior studies focused on the recovery after exercise [22], [24], [25], or used animal models [22], [23]. Exercise causes immediate perturbations of homeostasis that are gradually restored during recovery. Based on this notion, it can be suspected that changes in gene expression elicited by exercise gradually fade out during post-exercise recovery. In support, in our study a total of 938 genes was altered post-exercise in the exercising leg, whereas previously 173 genes were found to be increased 3 hours after termination of exercise and 37 genes 48 hours after exercise [24]. However, direct comparison is complicated by the use of different array platforms and statistical cut-offs. Mahoney et al. [24] used an custom made array, and only signal log ratios were available, which did not allow us to use the same statistical cut-off.

Many of the observed exercise-induced changes in gene expression are likely part of an acute stress response related to disturbances in homeostasis elicited by exercise. The most highly induced genes in the exercising leg were all members of the NR4A family, a subgroup of orphan receptors within the nuclear receptor superfamily. NR4A1 and NR4A3 have been reported to be upregulated shortly after acute exercise and during recovery in rat [40], [41], pig [23], and human [24], and this upregulation likely occurs locally by contractile stimuli [40]. This finding was confirmed by our study in which we observed an upregulation of NR4As in the exercising, but not in the non-exercising leg. NR4A transcription factors are also known to be induced by adrenaline and noradrenaline [39]. Circulating adrenalin and noradrenalin levels were increased in our study but must exert only a minor effect as NR4As were exclusively induced in the exercising leg,. Upregulation of NR4A2 has been observed once in human but its role during exercise is unknown [24]. NR4A1 and 3 are thought to play a key role in regulating energy metabolism and early adaptation [42], [43], [44]. Combined analysis of our study and the study of Mahoney et al. suggests that NR4A2 is induced immediately during exercise and decreases relatively quickly after exercise during recovery, whereas NR4A3 shows a different pattern characterized by a relative small induction during exercise followed by a large induction in the recovery phase (figure S4 [24]). The results may imply that NR4A family might play an important role in the regulation of metabolic responses after exercise.

Unlike in other subjects, in two subjects the overall magnitude of gene expression changes in the exercising and non-exercising leg were very similar. It is unclear how such an extreme response can occur in a resting leg that was not engaged in any concentric contractions. We cannot completely exclude these two subjects performed significant isometric contractions in the non-exercising leg, although subjects were instructed not to. Alternatively, a potential role for (involuntary) neural stimulation muscles may be envisioned. It is known that neural stimulation can induce gene expression changes via increased calcium concentrations in the skeletal muscle [45], [46], [47], as well as via other mechanisms [48]. Since energy utilization and metabolic flux must have been much lower in the non-exercising leg, which showed gene expression changes comparable to the exercising leg, it can be inferred that the majority of gene expression changes are unrelated to metabolic flux. The exception are genes induced similarly in the exercising and non-exercising leg in all subjects.

One-legged cycling is frequently used as a model to enable direct comparison between exercising and resting muscle. Earlier studies encountered problems with repeated biopsies, showing inflammatory gene expression changes induced by repeated biopsies [49]. Other studies showed no effect of repeated biopsies on gene expression [50]. The main difference between these studies is that the study that did show an effect of repeated biopsies on gene expression used one incision for all biopsies [49], whereas the other study showing no effect performed new incisions for each biopsies [50], analogous to our protocol. This indicates that separate incisions might be crucial for reducing the effect of repeated biopsies, which was also verified by the lack of inflammatory genes changed in the resting muscle in our study.

In conclusion, exercise has profound effects on gene expression in human skeletal muscle, not only in exercising muscle but also in resting muscle. The latter effects are likely mediated by changes in circulating factors such as FFA and may explain why muscles not involved in the exercise movement may undergo favourable adaptations in response to exercise.

## Supporting Information

Figure S1 **qPCR confirming microarray results of NR4A family, JUNB and FOS (N = 11): fold changes of both legs are displayed, which are calculated by dividing the post-exercise by the baseline sample for both legs.** Before expression values were normalized by the housekeeping gene GAPDH. *  =  p<0.01. Depicted is mean ± SEM.
(TIF)

Figure S2 **The 20 most highly induced genes in the non-exercising leg, including subjects 4 and 8: heatmap of the top 20 upregulated genes in the non-exercising (N = 9), left panel shows top 20 of the non-exercising leg, right the corresponding genes in the exercising leg.** Green is a signal log ratio of −3, red a signal log ratio of 3. Values are displayed per subject to visualize inter-individual differences. FC  =  fold change, *  =  p<0.05, ^#^  =  p<0.1 between exercising and non-exercising leg.
(TIF)

Figure S3 **Acute endurance exercise induces several genesets in both legs related to immune response, skeletal muscle hypertrophy and stress.** Venn diagram shows the overlap between the upregulated genesets in both legs. Next to the circles are the top 5 enriched genesets. Genesets depicted in bold are overlapping between exercising and non-exercising leg, whereas geneset with a normal font are unique for that leg. FDR  = 0.2, exercising leg N = 9, non-exercising leg N = 7.
(TIF)

Figure S4 **Combined time course of gene expression of selected genes of Mahoney et al. and this study: signal log ratios of selected genes (based on presence in datasets and expression) are displayed directly after exercise (this study) and 3 and 48 hours after exercise (Mahoney et al. 2005).**
(TIF)

Table S1 **List of differentially expressed genes at baseline (T0) in exercising and non-exercising leg.**
(PDF)

Table S2 **Induced sets of transcription factor targets.**
(PDF)
