# Supplementary figures and images for: Pronounced Effects of Acute Endurance Exercise on Gene Expression in Resting and Exercising Human Skeletal Muscle
Source: PLoS One. 2012 Nov 30;7(11):e51066. doi: 10.1371/journal.pone.0051066 (PMC3511348; doi:10.1371/journal.pone.0051066)

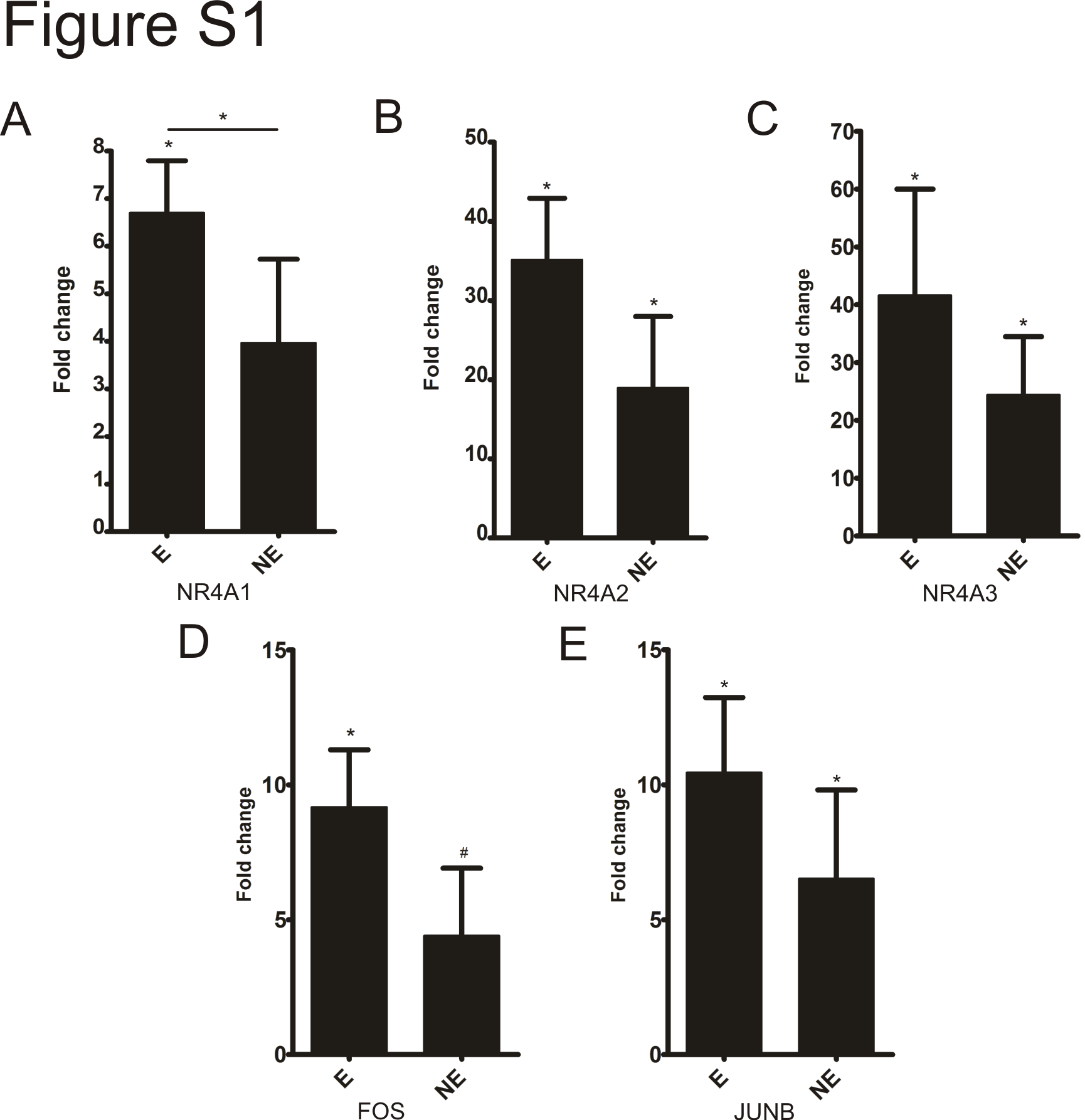

Supplement: Figure S1 — qPCR confirming microarray results of NR4A family, JUNB and FOS (N = 11): fold changes of both legs are displayed, which are calculated by dividing the post-exercise by the baseline sample for both legs. Before expression values were normalized by the housekeeping gene GAPDH. * = p<0.01. Depicted is mean ± SEM. (TIF) [file pone.0051066.s001.tif]

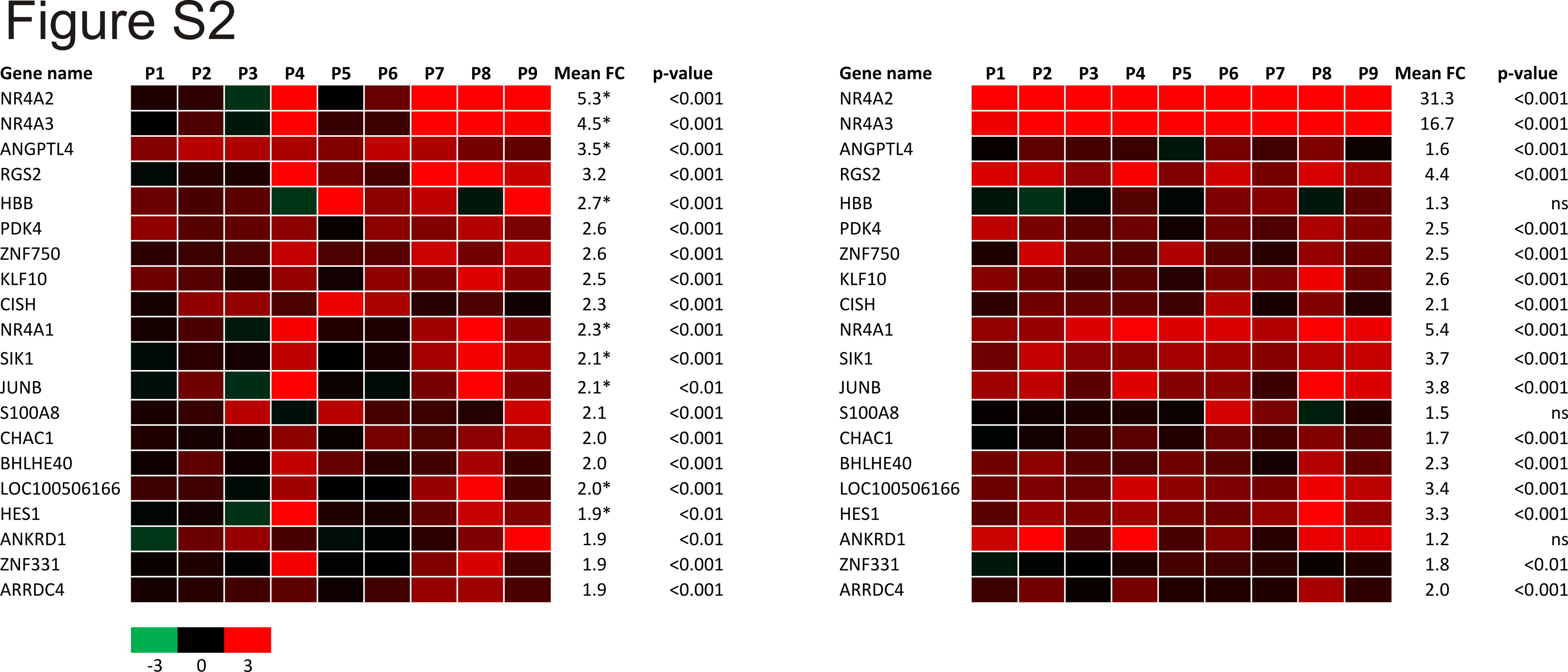

Supplement: Figure S2 — The 20 most highly induced genes in the non-exercising leg, including subjects 4 and 8: heatmap of the top 20 upregulated genes in the non-exercising (N = 9), left panel shows top 20 of the non-exercising leg, right the corresponding genes in the exercising leg. Green is a signal log ratio of −3, red a signal log ratio of 3. Values are displayed per subject to visualize inter-individual differences. FC = fold change, * = p<0.05, # = p<0.1 between exercising and non-exercising leg. (TIF) [file pone.0051066.s002.tif]

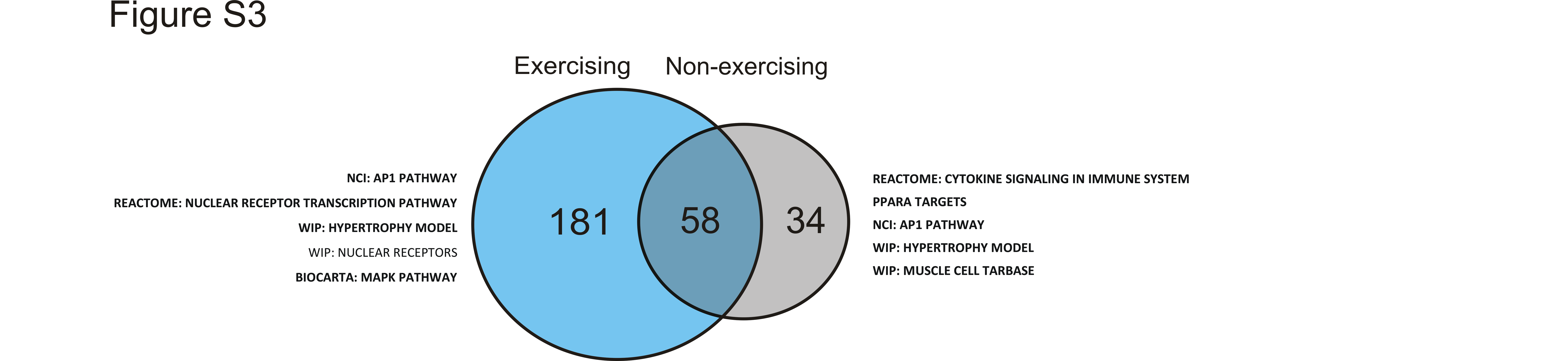

Supplement: Figure S3 — Acute endurance exercise induces several genesets in both legs related to immune response, skeletal muscle hypertrophy and stress. Venn diagram shows the overlap between the upregulated genesets in both legs. Next to the circles are the top 5 enriched genesets. Genesets depicted in bold are overlapping between exercising and non-exercising leg, whereas geneset with a normal font are unique for that leg. FDR = 0.2, exercising leg N = 9, non-exercising leg N = 7. (TIF) [file pone.0051066.s003.tif]

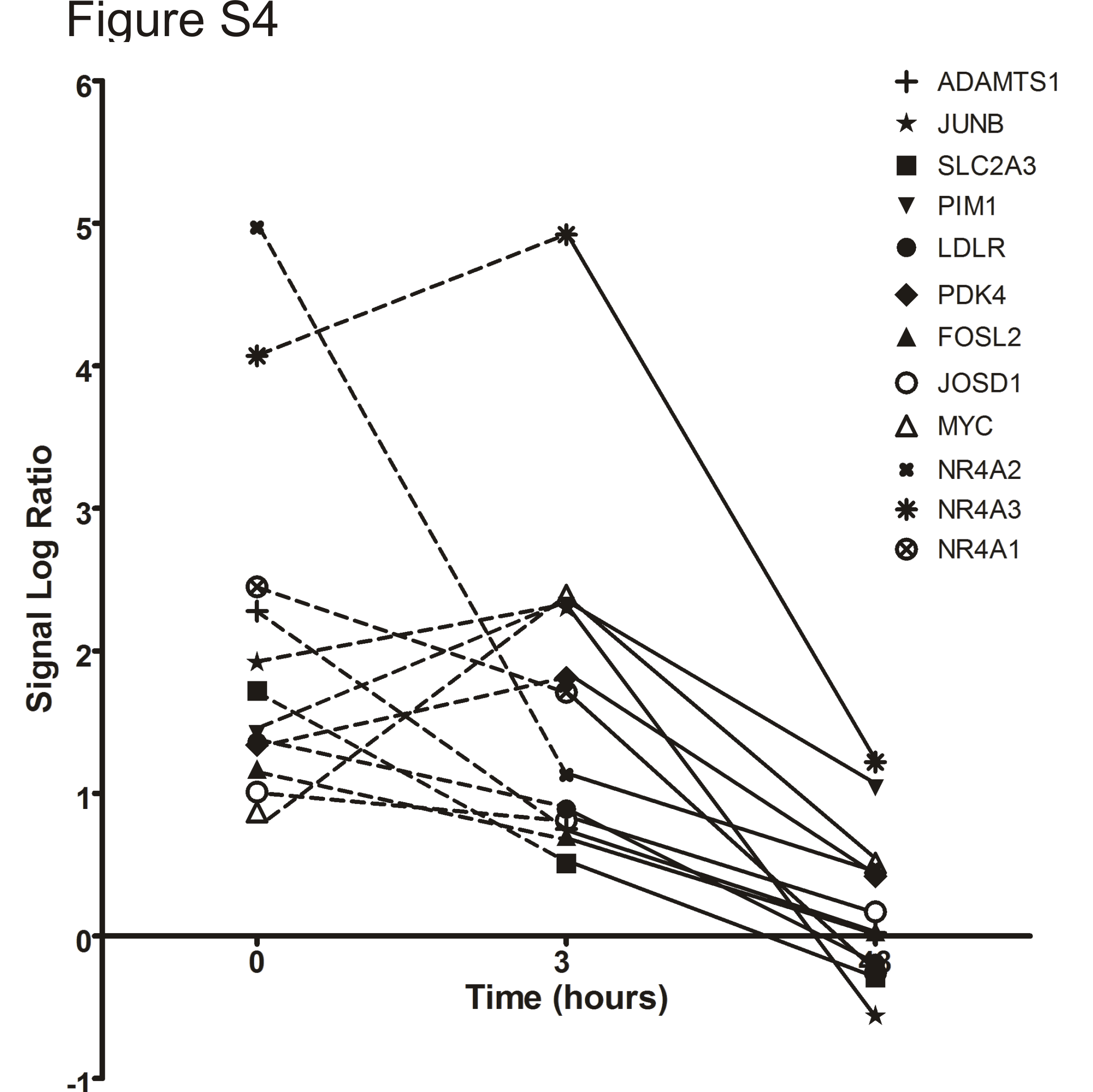

Supplement: Figure S4 — Combined time course of gene expression of selected genes of Mahoney et al. and this study: signal log ratios of selected genes (based on presence in datasets and expression) are displayed directly after exercise (this study) and 3 and 48 hours after exercise (Mahoney et al. 2005). (TIF) [file pone.0051066.s004.tif]
