# Supplementary material for: Pronounced Effects of Acute Endurance Exercise on Gene Expression in Resting and Exercising Human Skeletal Muscle
Source: PLoS One. 2012 Nov 30;7(11):e51066. doi: 10.1371/journal.pone.0051066 (PMC3511348; doi:10.1371/journal.pone.0051066)
Supplement: Table S1 — List of differentially expressed genes at baseline (T0) in exercising and non-exercising leg. (PDF) [file pone.0051066.s005.pdf]

Supplementary table 1 List of differentially expressed genes at baseline (T0) in exercising and non-exercising leg

Gene name	Mean FC	P value	FDR value
XPOT	-1.28545	0.0014	0.99962
PROCR	1.17887	0.0038	0.99962
C3orf71	1.191506	0.0049	0.99962
BANP	1.22251	0.0052	0.99962
MALL	1.283441	0.0088	0.99962
SPRR4	1.250174	0.0094	0.99962
LOC100505587	1.150362	0.0096	0.99962
THAP5	1.230518	0.0098	0.99962
