# Supplementary material for: Pronounced Effects of Acute Endurance Exercise on Gene Expression in Resting and Exercising Human Skeletal Muscle
Source: PLoS One. 2012 Nov 30;7(11):e51066. doi: 10.1371/journal.pone.0051066 (PMC3511348; doi:10.1371/journal.pone.0051066)
Supplement: Table S2 — Induced sets of transcription factor targets. (PDF) [file pone.0051066.s006.pdf]

Supplementary table 2 Induced transcription factor pathway and the genes contributing to this induction.

**Transcription factor**	**Target genes in dataset**
Exercising leg	
CREB1	APOLD1, ATF3, BHLHE40, BTG2, CEBPB, CIITA, CSRNP1, CYR61, DUSP14, EGR1, EGR2, ERRFI1, FLT1, FOS, FOSB, GADD45B, IDI1, IRS2, JUNB, LDLR, LSS, MCL1, MIDN, MKNK2, MYC, NAB2, NFIL3, NFKBID, NOS2, NR4A1, NR4A2, NR4A3, PDXK, PER1, PPARGC1A, PPP1R15A, RGS2, SERTAD1, SIAH2, SIK1, SLC2A3, VEGFA
SMAD3	BCL2L11, CCL2, CDKN1A, CTGF, ESR1, FOS, HEY1, JAM2, JUNB, JUND, MXD1, POR, RHOB, SKIL, SMAD1, SMAD7, TGFB1, TGFB3
SREBF1	ACLY, ADH1C, CDKN1A, CFD, FASN, HK2, HMOX1, HSPA1A/HSPA1B, HSPA5, IDI1, INSIG1, IRS2, LDLR, LGALS3, LSS, PIK3R3, SREBF1, TGFB1, VEGFA
STAT3	A2M, BCL2L11, BCL3, CCL2, CD9, CDKN1A, CIITA, CXCL2, DLL1, EGR1, FASN, FOS, GADD45A, ICAM1, IFI30, IFITM3, IL6R, JUNB, MCL1, MYC, NOS2, PCNT, PIM1, PPARGC1A, RORC, SGK1, SOCS3, SREBF1, STAT1, TAP1, TLR3, TNFRSF1B, VEGFA, WARS
HIF1A	ADAM17, ADM, AKAP12, AURKA, BIRC2, CDKN1A, CHKA, CXCL12, CYR61, ERO1L, FAM13A, FLT1, FOS, HK2, HMOX1, INHBB, IRS2, ITGA5, ITGB3, KDM3A, LIFR, MCL1, MYC, NDRG1, NOS2, NOS3, NOTCH1, NRARP, PDGFA, PDGFB, PFKFB3, SLC40A1, SMAD7, SOX9, TGFB1, TGFB3, VASP, VEGFA
FOXO1	ACLY, BCL2L11, CDC42EP3, CDKN1A, CTGF, ELOVL5, FASN, FOXO1, GADD45A, GADD45B, HSPA5 ,IER3, IRS1, IRS2, KLF2, LPL, ME1, PPARGC1A, SGK1, SLC25A1, SREBF1
EGR1	ATF3, CASP3, CASP9, CCL2, CDKN1A, CXCL2, FOSL1, GADD45A, GDF15, HMOX1, ICAM1, JUNB, JUND, ME1, MXD1, MYC, NAB2, NDRG1, PDGFA, PDGFC, TGFB1, THBS1, VEGFA
NOTCH1	CDKN1A, CEBPD, CFD, DLL1, FLT1, FOSB, HES1, HEY1, HEY2, ICAM1, MYC, NOS2, NR2F2, REL, RORC, TGFB1, TGFBR2, TP63
CLOCK	ANGPTL4, ARNT2, BHLHE40, BPHL, CBR1, CCRN4L, CHST3, DBP, DDIT3, DNAJB9, DUSP8, FASN, FKBP5, GADD45A, GADD45B, GDNF, GIMAP4, HSPA1A/HSPA1B, HSPH1, ICAM1, ID1, JMJD6, JUNB, KLF13, MAP2K3, MKNK2, NFIL3, PER1, PER2, POR, SLC20A1, ST3GAL5, TNFAIP1, TOR1B, USP2, WEE1
NR3C1	A2M, ABHD2, ABL1, ACTB, ACTN1, ADH1C, ADM, ANGPTL4, APOL3, AQP1, ARID4B, BAG2, BAG3, BCL2L11, BCOR, BHLHE40, BIRC2, BRAF, CASP9, CCL2, CDC42EP3, CDKN1A, CLK2, CSRNP1, CSRNP2, DAPK2, DAPK3, DDIT4, DDX5, DEDD2, DUSP1, DUSP16, DYNLL1, EGR1, EMP1, ERRFI1, FASTKD1, FKBP5, FOS, FOXO1, GADD45A, GADD45B, GLUL, HNRNPU, ICAM1, IER2, IER3, IGF1, ING2, IP6K3, JMJD6, JUND, LRRC8A, MCL1, MKNK2, MYC, NFKBIA, PARP4, PDE4B, PDGFA, PDPN, PIK3CB, PIK3R3, PLEKHF1, PPP1R13L, PPP1R15A, PPP2CA, PPP2R1B, RELT, RGS2, RHOB, RRAGC, SEMA3C, SERINC3, SERPINB9, SERTAD2, SGK1, SIAH1, SIAH2, SLC19A2, SLC38A1, SMAD1, SNAI2, STOM, THBD, TLE3, TNFAIP1, TNFAIP3, TNFRSF10B, TNFRSF12A, TNFRSF1B, TP53BP2, TSC22D3, UGCG, VGLL2, YWHAH
ATF4	ATF3, CDKN1A, CEBPB, CTH, DDIT3, DDIT4, ERO1L, GDF15, KLF4, LGALS3, MAP1LC3B, NDRG1, NID2, OSMR, PMP22, PYCR1, SLC7A5, SNAI2, TNFRSF12A, VEGFA, WARS
NR1H3	ACSL3, ARG2, FASN, LDLR, LPL, MYLIP, NFKBIA, NOS2, PPARGC1A, SREBF1
SREBF2	ACLY, CDKN1A, FASN, IDI1, INSIG1, IRS2, LDLR, LSS, SREBF1
FOXO4	BCL6, CDC42EP3, CDKN1A, CTGF, FASN, GADD45A, GADD45B, IDI1, IER3, SGK1
ELK1	CDKN1A, EGR1, EGR2, FOS, FOSL1, JAM2, JUNB, MCL1
ETS1	CDKN1A, CTGF, DIAPH1, FLT1, HSPA1A/HSPA1B, INSIG1, ITGB3, MCL1, MYC, NPR1, PLAU, PVR, SP100, ZNF302
FOXL2	ATF3, CXCL2, FOS, ICAM1, IER3, MAFF, NR4A3, PPARGC1A, PPP1R15A, RGS2, SMAD6, TNFAIP3
SMAD4	ANGPT2, CDC42EP3, CDK17, CDKN1A, CTGF, GADD45A, GADD45B, ID2, IER3, IRAK3, ITGA6, JAM2, MYC, POR, RASSF1, SERTAD1, SGK1, SLC6A1, SMAD3, SMAD6, SMAD7, TGFB1, THBS1, VEGFA
TP73	ABCC1, ADAM17, BHLHE40, CDKN1A, CTH, DBP, DDIT3, FASN, HIVEP1, IL4R, KLHL21, MIR22HG, PDGFB, PMP22, PTPN3, SAT1, TCEAL1, TGFB1, THBS1, TNFRSF1B, VEGFA
MYC	ADM, AKAP12, AMD1, ANGPT2, BIRC2, CDKN1A, CHKA, CLEC3B, COL15A1, COL4A1, COL6A3, COX7A2L, DDIT3, DUSP1, EIF4A1, FABP4, FASN, FBLN2, FBN1, GADD45A, GADD45G, GLS, HIST1H4A, HK2, HMOX1, HSP90AA1, HSPH1, ID2, ITGA6, KLF4, KRAS, MAT2A, MNT, MYC, MYO1C, ODC1, PFKP, PLAU, PLAUR, PMP22, SDCBP, SERINC3, SERPINH1, SGK1, THBS1, THBS2, TIMP2, TXNIP, VEGFA
Non-exercising leg	
PPARA	ANGPTL4, CES3, CPT1A, MVK, NR1D1, PDK4, PPM1D, RETSAT, SLC25A20, UCP2
